# Development and validation of the Emotional Climate Change Stories (ECCS) stimuli set

**DOI:** 10.3758/s13428-024-02408-1

**Published:** 2024-04-18

**Authors:** Dominika Zaremba, Jarosław M. Michałowski, Christian A. Klöckner, Artur Marchewka, Małgorzata Wierzba

**Affiliations:** 1 Laboratory of Brain Imaging, Nencki Institute of Experimental Biology, Polish Academy of Sciences, Warsaw, Poland; 2 Poznan Laboratory of Affective Neuroscience, SWPS University, Poznań, Poland; 3 Department of Psychology, Norwegian University of Science and Technology, NTNU, Trondheim, Norway

**Keywords:** Climate change, Pro-environmental behaviour, Emotion, Climate emotions, Valence, Arousal

## Abstract

Climate change is widely recognised as an urgent issue, and the number of people concerned about it is increasing. While emotions are among the strongest predictors of behaviour change in the face of climate change, researchers have only recently begun to investigate this topic experimentally. This may be due to the lack of standardised, validated stimuli that would make studying such a topic in experimental settings possible. Here, we introduce a novel Emotional Climate Change Stories (ECCS) stimuli set. ECCS consists of 180 realistic short stories about climate change, designed to evoke five distinct emotions—anger, anxiety, compassion, guilt and hope—in addition to neutral stories. The stories were created based on qualitative data collected in two independent studies: one conducted among individuals highly concerned about climate change, and another one conducted in the general population. The stories were rated on the scales of valence, arousal, anger, anxiety, compassion, guilt and hope in the course of three independent studies. First, we explored the underlying structure of ratings (Study 1; *n* = 601). Then we investigated the replicability (Study 2; *n* = 307) and cross-cultural validity (Study 3; *n* = 346) of ECCS. The collected ratings were highly consistent across the studies. Furthermore, we found that the level of climate change concern explained the intensity of elicited emotions. The ECCS dataset is available in Polish, Norwegian and English and can be employed for experimental research on climate communication, environmental attitudes, climate action-taking, or mental health and wellbeing.

**Supplementary Information:**

The online version contains supplementary material available at 10.3758/s13428-024-02408-1.

## Introduction

The climate crisis is recognised as the greatest threat that humanity has ever faced. The Intergovernmental Panel on Climate Change’s 2021 report clearly states that ‘it is unequivocal that human influence has warmed the atmosphere, ocean and land’. Widespread and rapid changes in the atmosphere, ocean, cryosphere and biosphere have occurred (IPCC, 2021). With the growing awareness of climate change and the associated risks, people start to experience intense emotions (Leiserowitz et al., 2021; Marczak et al., 2023; Marks et al., 2021; Zaremba et al., 2023). In turn, emotions shape one's attitudes and behaviour in the face of climate change, including climate change attitudes (Rode et al., 2021), climate change risk perception (van der Linden, 2015) and willingness to engage in climate change mitigation (Xie et al., 2019), as well as endorsement of climate policies (Bouman et al., 2020; Rees et al., 2015; Wang et al., 2018). According to the literature, factors such as gender, political views, understanding of climate change causes and impacts, social norms, value orientations and personal experiences with extreme weather events are all recognised as meaningful predictors of climate action (van der Linden, 2015). Affect and emotions stand out as one of the major determinants (Brosch, 2021). Negative affect toward climate change was found to be the single largest predictor of all examined cognitive, experiential and socio-cultural factors (van der Linden, 2015). In another meta-analysis, negative affect was identified as one of the largest predictors of climate action, along with descriptive norms, perceived self-efficacy and outcome efficacy (van Valkengoed & Steg, 2019). Interestingly, most of these findings come from qualitative and questionnaire studies. The results of experimental studies, however, often yielded inconclusive results (Brosch, 2021; Reser & Bradley, 2017; Schneider et al., 2021). The underlying cause can be attributed to differences in operational definitions and measurement of climate emotions (Chapman et al., 2017).

In the field of environmental psychology, researchers often investigate a wide variety of emotions related to climate change (henceforth: climate emotions), such as guilt, anger (Bissing-Olson et al., 2016; Harth et al., 2013), compassion (Swim & Bloodhart, 2015), anxiety (Whitmarsh et al., 2022) or hope (Bury et al., 2020). Each of these emotions is associated with a specific appraisal pattern and a specific context in which it is experienced (Lazarus, 1991). In fact, failure to provide consistent, replicable findings in this domain of research was mainly attributed to the lack of focus on specific appraisals that accompany the emotional experience of climate change (Brosch, 2021). Therefore, attempts were made to identify distinct climate emotions (Marczak et al., 2023; Pihkala, 2022; Zaremba et al., 2023) and corresponding appraisals, as well as to understand how they motivate behaviour (Harth et al., 2013; Landmann, 2021; Marczak et al., 2023; Zaremba et al., 2023). In the following paragraphs, we will briefly define emotions commonly identified as important motivators of climate action (Brosch, 2021; Reser & Bradley, 2017; Schneider et al., 2021; Shipley & van Riper, 2022). We also specify how the experience of each of these emotions translates to a certain behavioural tendency. In Table 1, we propose the formulation of cognitive appraisals related to the investigated climate emotions.

**Table 1:** Example of cognitive appraisals behind selected climate emotions

Emotion	Cognitive appraisal
Anger	‘Climate change is a result of harmful actions of individuals or institutions that I don’t identify with.’
Anxiety	‘Climate change is a real, urgent and severe threat to myself and to valued people and places.’
Compassion	‘Climate change results in undeserved and omittable suffering and harm to innocent beings.’
Guilt	‘Climate change is a result of my harmful actions or actions of groups that I identify with.’
Hope	‘Climate change can be limited with collective, coordinated, proactive efforts.’

In the context of climate change, **anger** can be understood as a moral outrage directed at individuals, people in power and institutions that deliberately and carelessly contribute to climate change (Marczak et al., 2023; Zaremba et al., 2023). In particular, it is experienced upon the realisation that climate change affects especially those who contribute the least to global emissions and those who will be most vulnerable to its consequences (Landmann & Hess, 2017). It increases arousal and activates behavioural tendencies to punish those blamed for causing climate change (Harth et al., 2013). The content of climate anger is relevant for the type of pro-environmental behaviours it promotes—anger at politicians and institutions predicts public sphere activism, while anger directed at general human qualities and actions predicts individual mitigation behaviours (Gregersen et al., 2023; Kleres & Wettergren, 2017; Stanley et al., 2021).

Climate **anxiety** is perhaps the most studied climate emotion and, as such, it has been operationalised in different ways. Some scholars frame it as disproportionate, debilitating, intense anxiety (Coffey et al., 2021) that results in active avoidance of the problem of climate change (Stanley et al., 2021). However, anxiety can also be defined as an adaptive reaction, in which climate change is perceived as a real, urgent and severe threat that needs to be addressed (Marczak et al., 2023; Zaremba et al., 2023). This type of anxiety is related to the action tendency to look for solutions and mitigate climate change (Pihkala, 2020). Thus, such anxiety can predict pro-environmental behaviours (e.g. Clayton & Karazsia, 2020; Helm et al., 2018; Hogg et al., 2021; Whitmarsh et al., 2022).

**Compassion** is experienced as one witnesses the suffering or undeserved harm of another being and results in the tendency to approach, help and support (Goetz et al., 2010; Landmann & Hess, 2017). The mobilising effect of compassion can be explained by the reduced psychological distance to climate change and its impact on all living things and beings (McDonald et al., 2015). It increases endorsement of climate mitigation efforts (Lu & Schuldt, 2016) and climate change activism (Swim & Bloodhart, 2015).

**Guilt** arises when one perceives their behaviour as incongruent with their moral standards (Tracy & Robins, 2007), or when one’s in-group is recognised as collectively responsible for causing harm (Wohl et al., 2006). It triggers action tendencies such as reparation and compensatory efforts (Harth et al., 2013; Parkinson et al., 2005; Smith & Ellsworth, 1985). In general, guilt predicts pro-environmental intentions and behaviours (Hurst & Sintov, 2022; Shipley & van Riper, 2022) and is an important moderator between climate change belief and mitigation behaviours (Ferguson & Branscombe, 2010).

In the case of **hope**, there is still considerable controversy, and researchers currently distinguish between the ‘false hope’ or denial-based hope, related to misguided beliefs about climate change, and the ‘constructive hope’, related to trust that climate change can still be halted with collective, coordinated efforts (Brosch, 2021). The latter type of hope was positively related to self-reported pro-environmental behaviour, support of specific policies and political engagement (Feldman & Hart, 2016, 2018; Ojala, 2015), and was shown to motivate climate action (Chadwick, 2015).

To date, the field of environmental psychology lacks ways to reliably elicit distinct climate emotions in experimental settings. Most previous studies used ad hoc stimuli, such as news reports (Chu & Yang, 2019; Nabi et al., 2018; O’Neill et al., 2013), photos (Gehlbach et al., 2022) or fictitious radio reports (Gustafson et al., 2020). While such stimuli are ecologically valid, they are difficult to use in experimental studies, in which strictly controlled conditions are required to establish a cause-and-effect relationship between variables. Therefore, there is a growing need for the development of validated emotional stimuli databases. To the best of our knowledge, there are only three available stimuli sets suitable for studying climate emotions. The first one, the Affective Climate Images Database (Lehman et al., 2019) comprises 320 pictures relevant to climate change. The second one, the Extreme Climate Event Database (EXCEED; Magalhães et al., 2018), consists of 150 pictures depicting natural disasters related to climate change. Finally, Climate Visuals (climatevisuals.org; Chapman et al., 2016) is a collection of pictures relevant to climate change selected based on both qualitative and quantitative criteria. Importantly, stimuli in the abovementioned datasets have been characterised only in terms of their dimensional properties, such as valence and arousal (Bradley & Lang, 1994; Stevenson et al., 2007), while in the context of climate change, there is a clear need to study discrete emotions (Barrett, 2006; Brosch & Steg, 2021). Empirical evidence demonstrates that it is important to control both dimensional and discrete properties of emotional stimuli (Briesemeister et al., 2014; Harmon-Jones et al., 2017).

Furthermore, despite the long tradition of using images in emotion research (Lang & Bradley, 2007; Marchewka et al., 2014; Michałowski et al., 2017), images may not always be well suited for the task of studying climate emotions. First and foremost, climate change is a multifaceted phenomenon that cannot be easily captured in a single frame. Images tend to be ambiguous, and it is therefore difficult to use them in emotion research while controlling for the associated appraisal pattern (Brosch, 2021). Finally, ecologically valid natural scenes are unsuitable for experimental designs that require strict control of physical properties (e.g. resolution, contrast, luminance). In contrast, textual stimuli (e.g. news reports, personal stories) have been used to convincingly describe the complex realities of climate change (e.g. Lu & Schuldt, 2015). Recent research suggests that naturalistic stimuli such as narratives are especially promising, as they provide all the relevant context and can be inspiring and deeply touching, thus inducing strong emotions (Goldberg et al., 2014; Saarimäki, 2021; Weber, 2006). Such naturalistic stimuli are frequently used in research, as they enable the study of many psychological processes, such as emotion (Jääskeläinen et al., 2021; Saarimäki, 2021), social perception (Mar, 2011; Redcay & Moraczewski, 2020) and language (Hamilton & Huth, 2020), in ecologically valid conditions. Despite their complexity, textual naturalistic stimuli can be easily edited (e.g. change of characters, change of cultural context) to meet the requirements of a particular study.

On the other hand, naturalistic stimuli pose many methodological challenges. Processing of complex textual stimuli demands active construction and simulation of the situation described in the story and involves many processes, such as linguistic processing, perspective taking, empathy, moral reasoning and autobiographical memory (Hsu et al., 2015). Thus, experiments using naturalistic stimuli are challenging to design and their results more difficult to interpret.

### Current study

In this article, we describe the development and validation of the Emotional Climate Change Stories (ECCS) stimuli set. ECCS consists of short naturalistic stories in which climate change is placed in the context of personal experience, rather than framed as an abstract scientific phenomenon (Harris, 2017; Morris et al., 2019; van der Linden et al., 2015). The stories were designed to elicit five distinct climate emotions—anger, anxiety, compassion, guilt and hope—all of which were indicated as motivating climate action. The ECCS set was developed in a series of studies conducted in Poland and Norway, two countries heavily dependent on fossil fuels, but with different policies towards achieving climate neutrality (Marczak et al., 2023; Zaremba et al., 2023). The validation procedure was conducted in line with previous studies (Bradley & Lang, 1994, 1999; Lang & Bradley, 2007; Marchewka et al., 2014; Wierzba et al., 2015). First, the ratings were collected in Poland from a large opportunity sample (Study 1, *n* = 601), as well as from an independent purposive sample with a demographic profile reflecting the population of Poland (Study 2, *n* = 307). This step was crucial to ensuring the high quality of ECCS ratings and investigating the replicability of the findings. Next, the ratings were collected in Norway from a purposive sample with a demographic profile reflecting the population of Norway (Study 3, *n* = 346) for the purpose of validating the ECCS ratings in a different culture.

## Method

### Materials

The stories in ECCS were inspired by the material collected in two qualitative studies conducted in Poland. In each case, we identified excerpts in which participants reported having experienced five emotion categories of interest: anger, anxiety, compassion, guilt and hope. In the first qualitative study (*n* = 40), we conducted semi-structured in-depth interviews with people strongly concerned about climate change (Zaremba et al. 2023). In this study, participants freely described a wide array of emotions experienced in the face of climate change and the context in which these emotions emerged. The collected material was analysed in a multi-step thematic analysis: the content of the interviews was carefully tagged by several researchers independently, and then used to identify recurring themes (e.g. drought, storm, heatwave, news in social media, politicians, talking to family). All the details can be found in the original work by Zaremba et al. (2023). In the second qualitative study (*n* = 523), we conducted a short online survey on a large opportunity sample. In this study, participants briefly described situations in which they specifically experienced anger, anxiety, compassion, guilt and hope in the context of climate change. The survey material was reviewed and tagged according to the thematic structure developed during the interview analysis (Zaremba et al. 2023). Based on the collected material (the qualitative interviews and the survey data), we generated several hundred short stories, representing the most commonly recurring themes. Finally, we selected 30 coherent and diverse stories representing each emotion category (150 stories in total). Furthermore, 30 neutral stories were developed on the basis of a standardised list of affect-related life events (Cohen et al., 2018).

The stories were professionally translated into Norwegian and English. Furthermore, the stories underwent proofreading to ensure they were adjusted to the cultural context, avoiding content that might be specific to a particular culture and could potentially be unfamiliar or less relevant in other cultural settings. Importantly, the ECCS stories are of similar length (Polish: *M* = 307.2, *SD* = 44.49; Norwegian: *M* = 295.2, *SD* = 49.35; English: *M* = 304.0, *SD* = 51.65). The full list of stimuli included in ECCS can be found in the Supplementary Materials. The most representative stories in each emotion category are presented in Table 2, and the mean story length (number of characters) for each language version of ECCS is summarised in Table S1.

**Table 2:** Stories that were found to best represent each emotion category

Type	Example story
Anger	Lisha is very rich and likes to change her wardrobe frequently. Ever since she has discovered a website with very cheap clothes, she orders 20–30 items a week. Some turn out to be a poor fit, so she throws them straight into the garbage bin. Still, Lisha does not consider this wasteful, because the clothes were ridiculously cheap.
Anxiety	Ada studies the oceans. Recently she had to double check her calculations, which indicated that melting Arctic ice may submerge areas inhabited by tens of millions of people. Unfortunately, her calculations turned out to be correct.
Compassion	It was a hot day when Emilie was on a bus with her seriously ill rabbit, taking it to the vet. The rabbit had trouble breathing, and the poor air conditioning was not able to cool the bus sufficiently. Before she could get to the vet, the rabbit died in its cage. Emilie got out, sat down at an empty bus stop and began to cry.
Guilt	By using plastic, we contribute to the growing environmental catastrophe every day. Even if we segregate waste carefully, there is no guarantee that plastic will be recycled. Patches of plastic garbage float on the surface of the oceans, the largest of which is three times the size of France.
Hope	Yoshida has discovered a new species of bacteria. The bacteria are able to break down plastic that would otherwise be deposited in landfills. Yoshida's discovery gained so much publicity that he was awarded another research grant. There are reasons to hope that his solution can be implemented on a large scale.
Neutral	Monica entered the room, opened the wardrobe and bent down to reach into one of the lower shelves. She took out her brown pants and put them on. Then she opened a drawer in her dresser and took out a belt. She passed it through the belt loops on her pants and fastened the buckle.

### Participants

Participants eligible to join the studies were native speakers of Polish (Study 1, 2) and Norwegian (Study 3). This criterion ensured that the participants had a solid understanding of their respective languages and minimised the possibility of inaccuracies or inconsistencies in the data obtained. Furthermore, we collected demographic data about participants’ gender, age, place of residence, education, parenthood status, occupation, climate activism and socio-economic status, as well as information about their belief in and concern about climate change (details provided in Table S2 of the Supplementary Materials).

### Study 1

A total of 749 Polish residents were recruited via advertisements on social media and through the SWPS University mailing list. After a data quality check, data from 601 individuals (504 women, 92 men and five non-binary persons) were retained. Depending on the recruitment platform, participants received no remuneration or were remunerated with student credit points.

### Study 2

A total of 349 Polish residents were recruited by a professional company. Purposive sampling was used in order to reach a diverse group of participants, with a demographic profile broadly reflecting the population of Poland. After a data quality check, data from 307 individuals (164 women, 142 men and one non-binary person) were retained. Participants received remuneration equivalent to €2.

### Study 3

A total of 450 Norwegian residents were recruited by a professional company. Purposive sampling was used in order to reach a diverse group of participants, with a demographic profile broadly reflecting the population of Norway. After a data quality check, data from 346 individuals (181 women and 165 men) were retained. Participants received remuneration equivalent to €0.50.

### Procedure

The procedures used in Studies 1–3 were as closely matched as possible. Participants completed the procedure remotely, working on their own devices (e.g. desktops, tablets, mobile phones). A purpose-built, secure web application was used to collect the ratings. The participants first read the description of the aims of the study, and provided their informed consent and demographic data. Then, participants were informed that they would be asked to read stories describing different situations and rate each of the stories on several scales. Participants provided their ratings using a slider from 0 to 99, with the bounds of the scales explicitly defined in the following way: valence (from *negative emotions*, through *no emotions*, to *positive emotions*); arousal (from *no arousal* to *strong arousal*); emotion categories: anger, anxiety, compassion, guilt, hope (from *not at all*, to *to a large extent*). The participants were able to return to the instruction screen at any time during the task. The stories to be rated in a given session were randomly selected from the initial pool of stories. However, stories with the smallest number of ratings collected so far had a greater chance of being selected. During the assessment task, each trial began with a display of a story in full-screen mode. Next, on the following screen, the participants were still able to see the story in the upper part of the screen, but this time, they were asked to rate the story in terms of valence, arousal, as well as the extent to which the story elicited anger, anxiety, compassion, guilt and hope. As soon as all the responses were submitted, the next trial would begin. There was no time limit to complete the task, but the time spent on reading the story and the time spent providing the ratings was recorded. After completing the ratings for 10 stories, participants could choose to continue rating additional stories or finish the task.

### Data preprocessing

To ensure data quality, we defined the following criteria: (1) only adult, native Polish speakers (in the case of Study 1 and Study 2) /native Norwegian speakers (in the case of Study 3) were permitted to join the study; (2) their responses regarding gender, age and the level of climate change concern had to be consistent with their responses to the same questions in the screening survey (applicable to Study 2 and Study 3 only); (3) participants had to complete ratings for at least 10 stories.

## Results

Here, we will use the following abbreviations to denote story types: ANG, anger; ANX, anxiety; COM, compassion; GUI, guilt; HOP, hope; NEU, neutral. Otherwise, we will use full terms to denote rating scales: valence, arousal, anger, anxiety, compassion, guilt and hope.

### General information about the collected ratings

A complete list of stories in all available language versions (Polish, Norwegian and English), together with their mean ratings, can be found in the Supplementary Materials. On average, each story was rated by 147.6 people (min = 141, max = 155), and depending on the study, a participant rated on average 19.93–23.41 stories. Depending on the study, the mean story presentation time was 6.8–11.5 seconds, and the mean story evaluation time was 16.4–19.5 seconds. A detailed report on the total number of ratings and the number of stories rated per participant in each study can be found in Table S3 in the Supplementary Materials.

### Dimensional characteristics

**Fig. 1:**
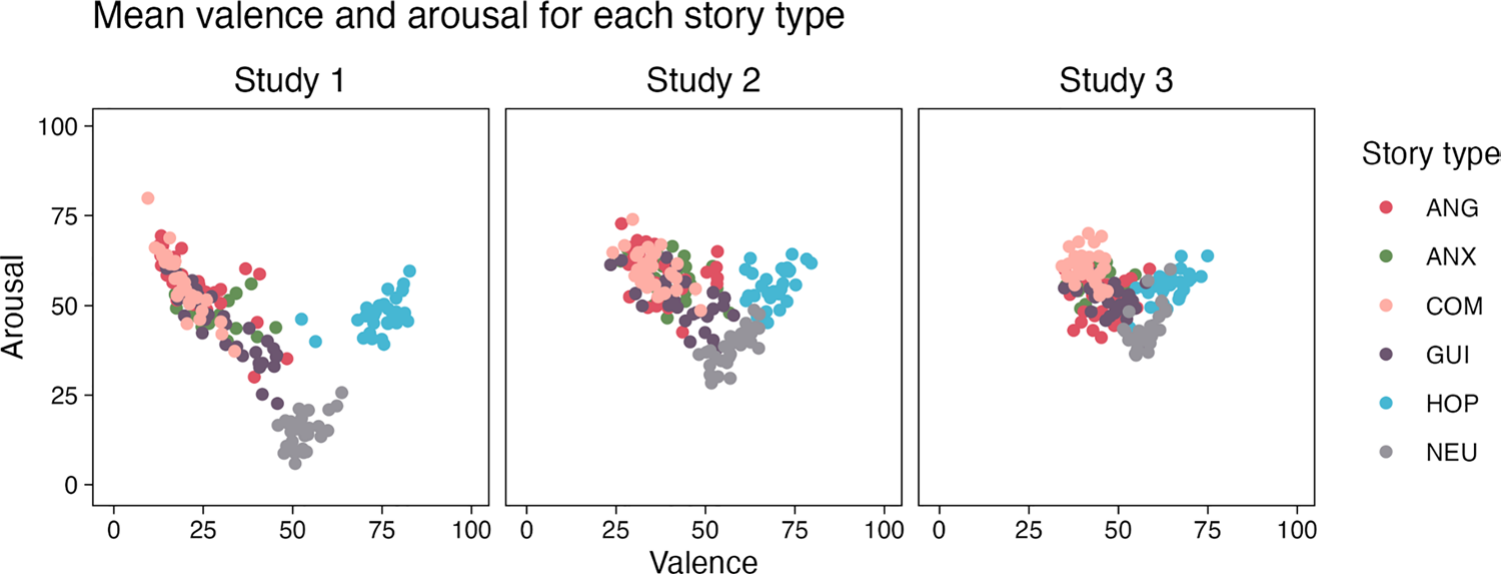
Mean valence and arousal ratings for each story. *Note:* Individual stories are represented by dots. Colours denote different story types: ANG - anger, ANX - anxiety, COM - compassion, GUI - guilt, HOP - hope, NEU - neutral

First, we investigated the distribution of valence and arousal ratings, which are commonly used to characterise emotional stimuli (e.g. Lang & Bradley, 2007; Lehman et al., 2019; Magalhães et al., 2018; Marchewka et al., 2014; Wierzba et al., 2015, 2022). Figure 1 presents the pattern of results obtained in Studies 1–3. In agreement with previous research, we observed a non-linear relationship between valence and arousal ratings. Furthermore, three characteristic clusters emerged: stories evoking negative emotions (ANG, ANX, COM and GUI stories, characterised by high arousal and low valence), neutral stories (NEU stories characterised by low arousal and moderate valence) and stories evoking positive emotions (HOP stories, characterised by high arousal and high valence). Thus, stimuli rated more extreme in terms of valence (either more negative or more positive) were also rated high in terms of arousal. Although the dispersion of ratings in the dimensions of valence and arousal was greater in Study 1 than in Studies 2 and 3, the general pattern of results seems consistent across the studies. Comparisons of ratings of each story between studies can be found in Figures S1 and S2 in the Supplementary Materials.

### Climate emotions

**Fig. 2:**
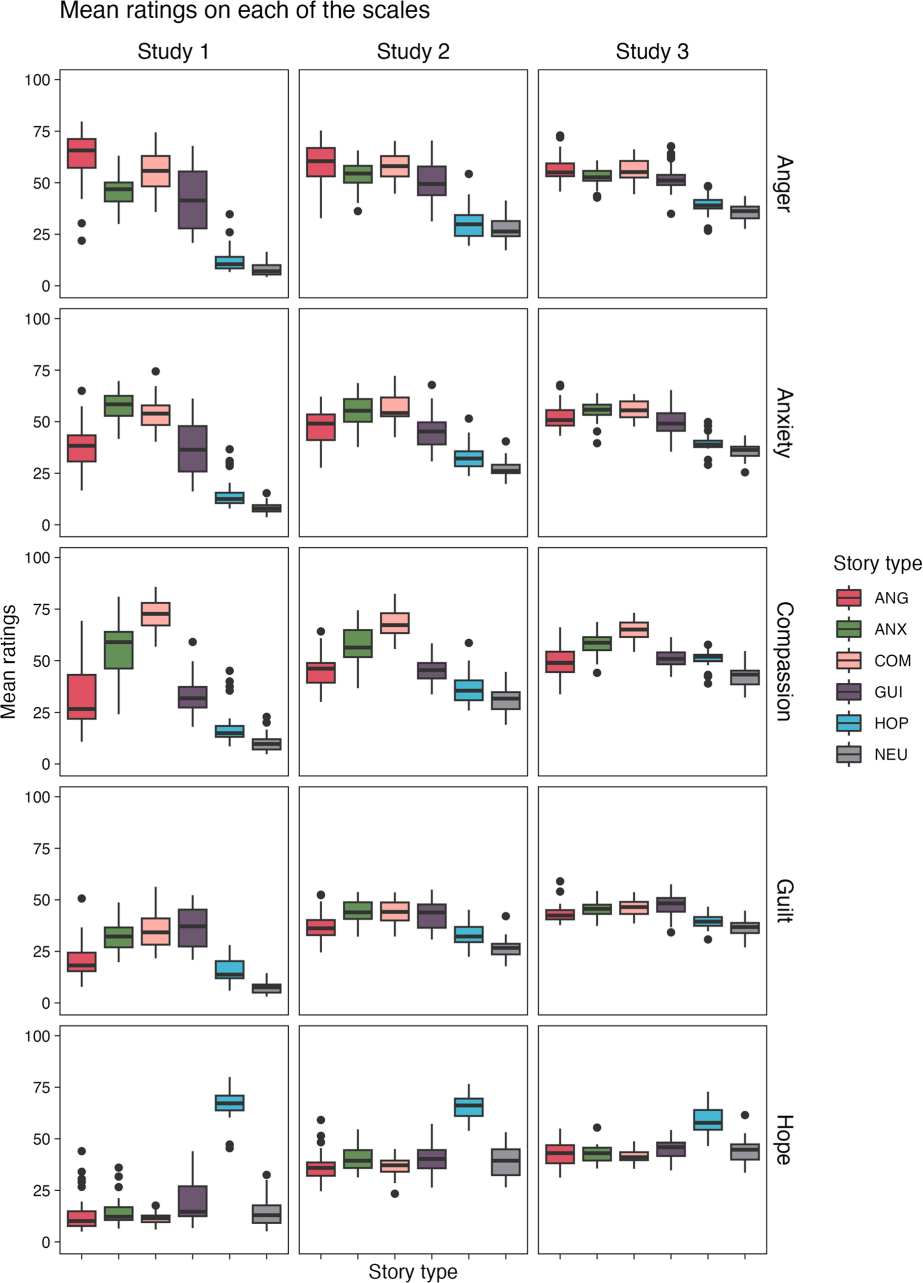
Distribution of mean anger, anxiety, compassion, guilt and hope ratings for each story type. *Note:* The results are presented separately for each rating scale (rows) and each study (columns). Colours denote different story types: ANG - anger, ANX - anxiety, COM - compassion, GUI - guilt, HOP - hope, NEU - neutral

Next, we explored the distribution of ratings in terms of distinct climate emotions: anger, anxiety, compassion, guilt and hope. The general distribution of mean ratings for each rating scale and each study is presented separately in Fig. 2.

Furthermore, we statistically examined the effects of story type and study on the mean ratings of anger, anxiety, compassion, guilt and hope. In particular, we were interested in determining whether stories belonging to a specific story type were rated similarly on the scale relevant for that story type (e.g. anger ratings for ANG stories, anxiety ratings for ANX stories). As the initial step, we performed a MANOVA with story type and study as factors. We found significant main effects for story type, *F*(5, 35,050) = 331.05, *p* < .001, Pillai’s trace = .955, and study, *F*(2, 14,014) = 144.26, *p* < .001, Pillai’s trace = .187, revealing that both factors had a significant overall impact on the collected ratings. Furthermore, a significant interaction effect was observed, *F*(10, 35,050) = 36.61, *p* < .001, Pillai’s trace = .248). To further investigate this interaction, ANOVA analyses were performed for each rating scale separately and corrected for multiple comparisons. In each case, the ANOVAs were followed by post hoc comparisons to further investigate whether the ratings collected in the compared studies differed. For simplicity, we focused on comparing ratings on story-type relevant scales.

First, we compared samples with different motivations to participate in the study (Study 1: opportunity sample, Study 2: purposive sample). The ANOVAs (with a correction for multiple comparisons) revealed a significant interaction effect between story type and study for all of the rating scales (anger: *F*(5, 5084) = 18.44, *p* < .0001, partial η^2^ = .02; anxiety: *F*(5, 5084) = 17.14, *p* < .0001, partial η^2^ = .02; compassion: F(5, 5084) = 30.012 *p* < .0001, partial η^2^ = .03; guilt: *F*(5, 5084) = 7.32, *p* < .0001, partial η^2^ =.01; hope *F*(5, 5084) = 35.87 *p* < .0001, partial η^2^ = .03). Post hoc comparisons showed that none of the interactions between story type and study were significant for the relevant scales (ANG stories on anger scale: *p* = .64; ANX stories on anxiety scale: *p* = .93; COM stories on compassion scale: *p* = .16; GUI stories on guilt scale: *p* = .07; HOP stories on hope scale: *p* > .99), indicating no significant differences in how participants from the opportunity and purposive samples rated stories of each type on the relevant scales (see Table S4 in the Supplementary Materials).

Next, we compared story ratings between samples from different countries, where different language versions of ECCS were used (Study 2: Polish sample, Study 3: Norwegian sample). The ANOVAs (with a correction for multiple comparisons) revealed a significant interaction effect between story type and study for almost all rating scales (anger: *F*(5, 3611) = 8.99, *p* < .0001, partial η^2^ = .01; anxiety: *F*(5, 3611) = 4.09, *p* = .015, partial η^2^ = .01; compassion: *F*(5, 3611) = 16.28, *p* < .0001, partial η^2^ = .02; guilt: *F*(5, 3611) = 2.15, *p* = .84, partial η^2^ < .01; hope *F*(5, 3611) = 5.97, *p* < .0001, partial η^2^ = .01). Similarly, post hoc comparisons showed that none of the interactions between story type and study were significant for the relevant scales (ANG stories on anger scale: *p* > .99, ANX stories on anxiety scale: *p* > .99, COM stories on compassion scale: *p* = .86, GUI stories on guilt scale: *p* = .33, HOP stories on hope scale: *p* = .19), indicating no significant differences in how participants from Poland and Norway rated stories of each type on relevant scales (see Table S5 in the Supplementary Materials).

### Impact of climate change concern on story ratings

Finally, we investigated whether the level of concern about climate change predicts the intensity of emotions experienced when reading the ECCS stories. To start, we divided our sample into three groups (of approximately equal size), representing three levels of concern about climate change: *low* (*n* = 395; score of 1–3 on a five-point scale), *medium* (*n* = 500; score of 4 on a five-point scale) and *high* (*n* = 343; score of 5 on a five-point scale). Next, for each participant, we calculated their summary emotion score, representing the intensity of experienced emotions. The participant’s summary score was calculated for each story type separately, using the following formula:

$$score = \left|{r}_{valence}- 50\right|*{r}_{arousal} ,$$

where $r$ denotes the mean ratings for a given story type. The summary score was then rescaled to take values between 0 and 1.

**Fig. 3:**
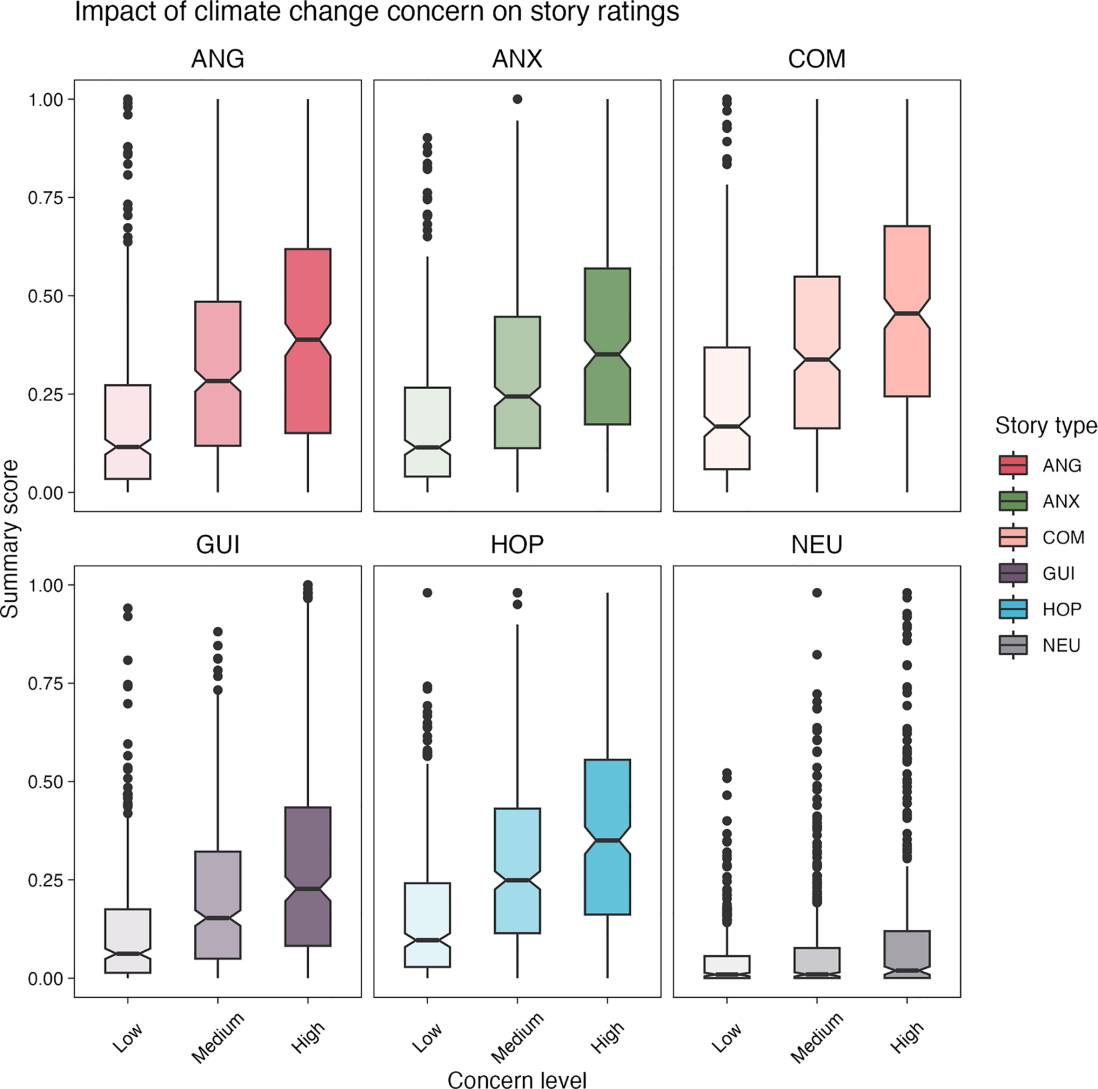
The impact of climate concern on story ratings. *Note:* For simplicity, we recoded the climate change concern from a five-point to a three-point scale (*low*, *medium*, *high*). Colours denote different story types: ANG - anger, ANX - anxiety, COM - compassion, GUI - guilt, HOP - hope, NEU - neutral

The relationship between the summary emotion score and the level of climate change concern for each story type is shown in Fig. 3. To test this relationship statistically, we performed a linear regression analysis. The model was statistically significant, *F*(2, 6933) = 329.85, *p* < .001, suggesting that the collected ratings could be predicted based on participants’ climate change concern. However, the model accounted for a rather weak proportion of variance (*R*^2^ = 0.09, adj. *R*^2^ = 0.09). Results showed that participants with medium climate concern had significantly higher scores than those with low concern (β = 0.10, 95% CI [0.09, 0.12], *t*(6933) = 15.37, *p* < .001), as did participants with high climate concern (β = 0.19, 95% CI [0.17, 0.20], *t*(6933) = 25.54, *p* < .001).

### Classification of stories into emotion classes

Previous research has demonstrated that the emotional response to climate change is complex and multifaceted. Similarly, we expected that realistic personal stories about climate change would evoke a range of different emotions simultaneously. For instance, a story about trees damaged by municipal workers could elicit feelings of compassion towards the victims (the trees) and anger towards those responsible for causing the harm (the people in power who issued a permit to cut the trees). Each individual ECCS story was rated according to the intensity of five emotions: anger, anxiety, compassion, guilt and hope. In other words, some stories could be associated mostly with one dominant emotion (e.g. hope), while others could be related to several emotions (e.g. anxiety and compassion) or none.

We used the collected data to identify stories that best represented each emotion category. To achieve this, we adopted a method introduced in our previous work (Wierzba et al., 2015, 2022). Here, we consider a five-dimensional hypercube, with each axis corresponding to one of the emotions. The ratings of a given story determine its position in the hypercube. Five of the hypercube's corners represent the emotion classes: [99 0 0 0 0] anger, [0 99 0 0 0] anxiety, [0 0 99 0 0] compassion, [0 0 0 99 0] guilt and [0 0 0 0 99] hope. The origin, namely [0 0 0 0 0], represents the neutral class. The distance of each story from each of the corners can be calculated using the standard formula:

$$d(p,q) =\sqrt{{( p}_{1}-{q}_{1} {)}^{2}+{( p}_{2}-{q}_{2} {)}^{2}+ ... +{( p}_{k}-{q}_{k} {)}^{2}}$$

The distances are first calculated for each participant separately, based on the individual ratings contributed by each person. Next, the distances are averaged over all participants to yield a summary measure of the distance of each story from each of the corners:

$$\overline{d}=\frac{1}{n}\left({\sum }_{i=1}^{n}{d}_{i}\right)=\frac{{d}_{1}+{d}_{1}+ ... +{d}_{n}}{n}$$

The following conditions must be fulfilled for a story to be assigned to one of the classes: (1) the story’s distance to the respective class must be smaller than a chosen threshold; (2) the story must meet the first condition for one class only; (3) if the story falls within an area of intersection of two (or more) classes, it remains unclassified; (4) if the story does not meet the first condition for any of the classes, it remains unclassified; (5) the assigned class must match the initial category label (i.e. the emotion the story was constructed for).

Importantly, this method can be tailored to one's needs. One approach is to set a threshold value for each class, which will determine the size of each class (i.e. the number of stories it contains). Alternatively, one could set a desired class size for each class, which would require a particular combination of threshold values to achieve.

First, we tested different values of thresholds by simultaneously increasing the threshold for each of the classes. Figure S3 in the Supplementary Materials illustrates how class sizes change as we gradually increase the thresholds from 0 to 140 (the minimum and maximum possible distance between the hypercube's corners, respectively). We observed that it was especially easy to classify stories into the neutral class, followed by the hope, anger and compassion classes. Almost no stories were classified into the anxiety class, and none were assigned to the guilt class.

**Fig. 4:**
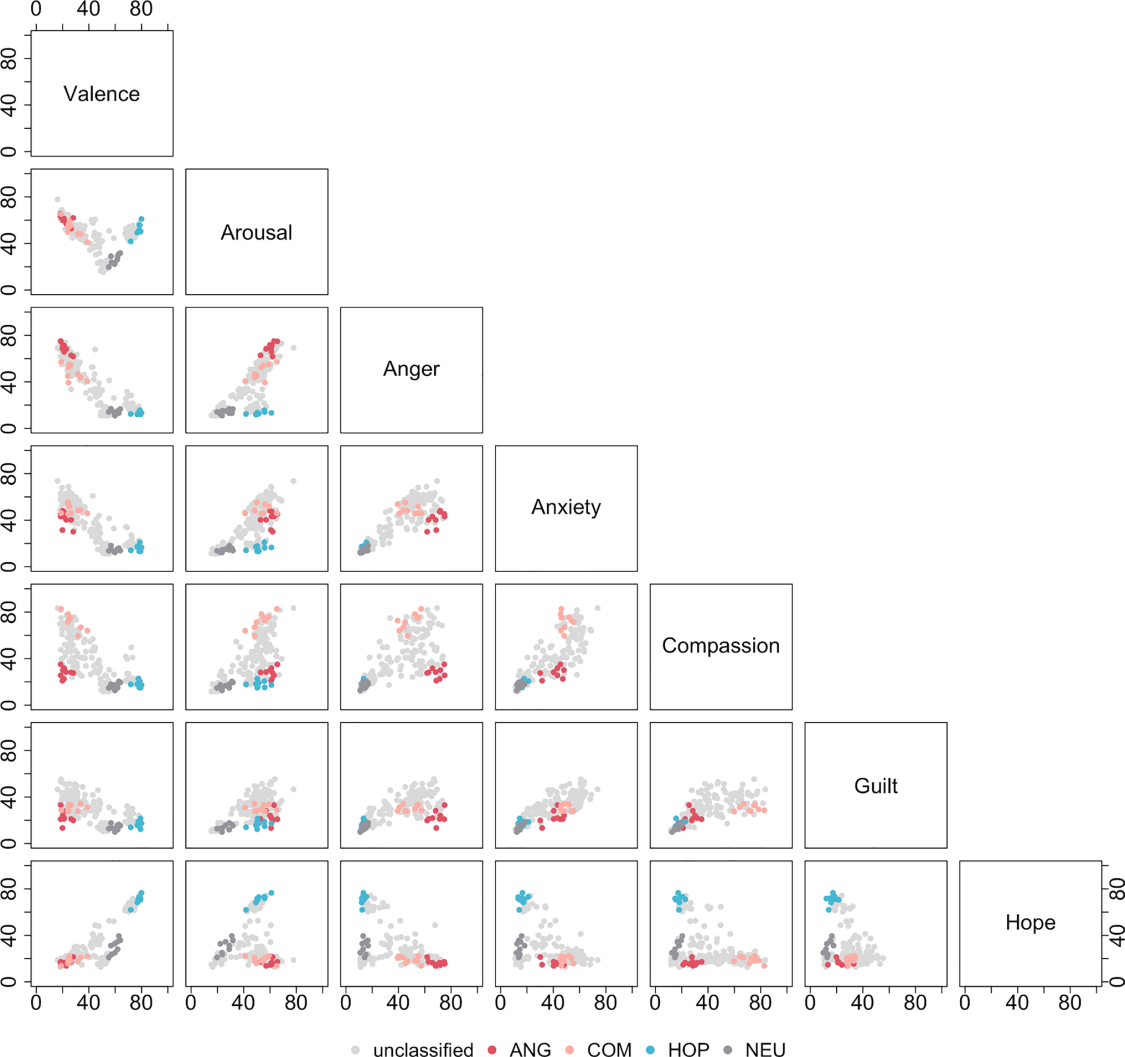
Distribution of the ratings of stories from ANG, COM, HOP and NEU classes identified with the classification algorithm. *Note:* Here, rows and columns represent different rating scales: valence, arousal, anger, anxiety, compassion, guilt, and hope. Each subplot shows the distribution of the ECCS stories in the domain of two rating scales. For instance, the top-left corner shows the distribution of the stories in the domain of valence (horizontal axis) and arousal (vertical axis). Each dot represents one of 180 stories, with colours denoting different classes identified with the classification algorithm: ANG - anger, COM - compassion, HOP - hope, NEU - neutral. Unclassified stories are marked in light grey for visualisation purposes

Based on these findings, we attempted to create NEU, HOP, ANG and COM classes of equal size. The threshold values were determined with the help of a simple genetic algorithm (the exact implementation of the algorithm is available in the Supplementary Materials). Figure 4 presents the resulting distribution of classes, each containing nine stories. The list of classified stories can be found in the Supplementary Materials.

## Discussion

Recent findings indicate that emotions play a crucial role in shaping people's perception of climate change. While most of these findings come from qualitative and questionnaire studies, experimental studies are of particular importance as they allow us to establish causal relationships between the studied variables. Thus, there is a growing need for the development of validated emotional stimuli databases, suitable for reliably and effectively eliciting distinct emotions related to climate change in experimental settings. The employment of such validated stimuli sets, in contrast to ad hoc stimuli, enables researchers to exert greater control over their experiments, thereby promoting a higher level of objectivity in research inferences.

In this article, we describe the development and validation of the Emotional Climate Change Stories (ECCS) database. ECCS is a collection of naturalistic, relatable stories that place climate change in the context of personal experience, thus avoiding the framing of this phenomenon solely as a distant and abstract scientific fact (Morris et al., 2019; Weber, 2006). For instance, some of the stories describe people who learn about the consequences of climate change and make personal choices that prioritise the environment (Ockwell et al., 2009). This format has been confirmed as an effective means of eliciting engagement in climate communication research (Gustafson et al., 2020; Jones & Song, 2014; Moezzi et al., 2017). Importantly, the stories were created based on qualitative in-depth interviews (Zaremba et al., 2023), in which individuals freely described a wide array of emotions experienced in the face of climate change and the context in which these emotions emerged.

The data-driven, narrative origins of ECCS stories allow for their analysis through the lens of empirical ecocriticism (Schneider-Mayerson et al., 2020). Even though ECCS are shorter than most texts investigated by ecocritics (novels, poetry, children's literature, film, etc.), they definitely reflect contemporary discourse on climate change. Future research could use this approach to provide additional insights.

The ECCS stories are characterised in terms of their dimensional properties, such as valence and arousal (Bradley & Lang, 1994; Stevenson et al., 2007), as well as in terms of discrete emotions (Barrett, 2006; Ekman, 1992). Moreover, we demonstrate that the story ratings can be predicted by the self-reported level of concern about climate change. This relationship was consistently observed for all story types except the neutral stories, providing evidence for the validity of the collected ratings. In particular, our findings are in line with the notion that individuals strongly concerned about the environment should—through their personal, lived experiences of climate change—find the stories more relatable and thus report higher intensity of emotions (Morris et al., 2019). Interestingly, although investigated, this relationship was not observed for other existing datasets of stimuli related to climate change. Specifically, Lehman and colleagues (Lehman et al., 2019) examined whether the valence and arousal ratings of climate change images depended on participants’ environmental attitudes, but found no evidence for that.

As for the dimensional properties of ECCS, we observed a non-linear relationship between valence and arousal. Specifically, stories that were rated more extreme in terms of valence (either more negative or more positive) were also rated as more arousing. This finding is in agreement with many previous studies (Lang & Bradley, 2007; Magalhães et al., 2018; Marchewka et al., 2014; Riegel et al., 2015; Wierzba et al., 2015). Furthermore, negative stories were rated more arousing than positive stories. Similarly, previous studies demonstrated that negative, disturbing pictures of climate change were found most salient (Lehman et al., 2019; Leiserowitz, 2006).

As for the discrete properties of ECCS, we were able to identify stories that predominantly represent one emotion category, such as anger, compassion or hope. ECCS contains stories representing some of the most studied climate emotions: guilt, anger (Bissing-Olson et al., 2016; Harth et al., 2013), compassion (Swim & Bloodhart, 2015), anxiety (Whitmarsh et al., 2022) or hope (Bury et al., 2020). Each of these emotions is associated with a specific appraisal pattern and a specific context in which it is experienced (Brosch, 2021), and in turn can lead to different behavioural tendencies (Böhm, 2003; Landmann, 2021; Marczak et al., 2023; Zaremba et al., 2023). Individuals from different populations did not differ in terms of how they rated stories on relevant scales (e.g. ANG stories on the anger scale). However, they did differ in terms of how they evaluated stories on the remaining scales (e.g. ANG stories on the compassion scale). In particular, we observed significant differences between Studies 1 and 2 (same country, different motivation to participate) in this regard. In Study 1, both the range and the variance of responses was greater than in Study 2. This suggests that populations with different demographic profiles (e.g. young, mostly student sample with non-financial motivation to participate vs general population with financial motivation to participate) might show a slightly different response pattern. Interestingly, ratings collected in Studies 2 and 3 (same motivation to participate, different country) were much more similar, suggesting that the collected ratings may be universal across the context of the Global North. Overall, our results—replicated across different populations and different cultures—suggest that by framing climate change in a specific context, one can reliably elicit distinct climate emotions.

At the same time, our results clearly indicate that ECCS stories differ in their potential to represent the emotion category they were intended to evoke. In other words, some stories convey a single, pure emotion, while others elicit a blend of emotions. With the use of the classification method described in the manuscript, we were able to identify stories related strongly and specifically to anger, compassion and hope, but we failed to identify stories related to anxiety and guilt. ANX stories received comparably high anxiety and compassion ratings, while also scoring relatively high on anger and guilt scales. These results are in line with previous findings, which suggest that these emotions often co-occur (Hatfield et al., 2009; Marczak et al., 2023). Evoking strong and specific guilt turned out to be especially challenging. Cognitive appraisals related to the guilt category revolve around the locus of responsibility for taking part in causing and solving climate change (Wang et al., 2018; Zaremba et al., 2023). GUI stories were designed to evoke guilt by promoting identification with the character or the group that contributes to climate change. They were, however, associated with the least specific emotional response and the lowest overall intensity of emotion compared to other stories. On average, elicited feelings of guilt were not intense. Perhaps the fact that most of the ECCS stories were third-person narratives makes it a less suitable means of eliciting guilt, for example in comparison to the Ecological Footprint Task (Mallett et al., 2013). Furthermore, people demonstrate resistance in response to attempts to evoke climate emotions that threaten self-esteem or sense of security (Ma & Hmielowski, 2022). The low intensity of reported guilt might have also resulted from the fact that the participants tried to preserve a positive self-image (Caillaud et al., 2016) and therefore used a variety of coping strategies (such as disidentifying with the protagonist or minimising the negative consequences of climate-unfriendly actions). Similarly, climate anxiety may also activate maladaptive emotion-focused coping strategies, such as denial or de-emphasising the threat (Haltinner & Sarathchandra, 2018; Ojala, 2012). Masking climate emotions as a form of defensive reaction to cognitive dissonance or a threat to self-concept has been identified in previous studies (Bercht, 2021). We advise taking these factors into consideration when using ECCS for research purposes.

The ECCS dataset was created based on the data collected in Poland and Norway, two European countries that belong to the Global North and are highly reliant on fossil fuels (Brauers & Oei, 2020; EIA, 2019). While we created ECCS with the aim that the stories would be as culturally universal as possible, we acknowledge that the stories are more representative of the experiences of people living in the Global North countries. Because the relatability of such stories may depend on the geopolitical context, we encourage researchers who plan further ECCS adaptations to take these limitations into account. Moreover, in-depth interviews and survey studies that inspired the stories were conducted between 2020 and 2021. Thus, ECCS narratives describe contemporary discourse on climate change (e.g. technological solutions to climate change that are presently being developed; political and social issues that are currently prevalent in traditional and social media). Over time, as the perception of climate change and its impacts evolves, the stories may become less relevant or relatable.

## Future directions

The ECCS database holds the potential for facilitating research across various disciplines of scientific study. In particular, it can be used to explore the role of emotions in motivating pro-environmental behaviour, policy support and consumer decisions, as well as psychological well-being and mental health. The collected ratings turned out to be highly consistent across different populations and different cultural contexts, which suggests that ECCS can be successfully used in various cultural contexts (Henrich et al., 2010). Importantly, the ratings are available both as summary scores and as individual scores to enable research on specific groups, such as different generations (Gray et al., 2019), parents (Schneider-Mayerson & Leong, 2020) or climate activists (Eide & Kunelius, 2021). Consequently, researchers can flexibly choose stimuli with desired parameters that best suit their needs. The choice of emotion categories in ECCS was based on our focus on the role of emotions in motivating pro-environmental behaviour. For this reason, our research was not concerned with many other climate emotions, which are currently widely recognized by the research community. In future, it may be useful to extend the ECCS dataset, by including stories representing other climate emotions. Because the ECCS stories have only been investigated with self-report measures, it would also be beneficial to investigate their properties using more objective measures (e.g. physiological reactions, brain activity). The ECCS stories, together with the accompanying data and code used for the analysis, are publicly available for scientific, non-commercial use and can be found at: https://osf.io/v8hts/.’

### Supplementary Information

Below is the link to the electronic supplementary material.

Supplementary file1 (DOCX 658 KB)

## Data Availability

Data and materials can be accessed at: https://osf.io/v8hts/

## Code availability

Code used for the analyses can be accessed at: https://github.com/nencki-lobi/ECCS
