# Correction: Development and validation of the Emotional Climate Change Stories (ECCS) stimuli set

**DOI:** 10.3758/s13428-024-02460-x

**Published:** 2024-06-21

**Authors:** Dominika Zaremba, Jarosław M. Michałowski, Christian A. Klöckner, Artur Marchewka, Małgorzata Wierzba

**Affiliations:** 1 Laboratory of Brain Imaging, Nencki Institute of Experimental Biology, Polish Academy of Sciences, Warsaw, Poland; 2 Poznan Laboratory of Affective Neuroscience, SWPS University, Poznań, Poland; 3 Department of Psychology, Norwegian University of Science and Technology, NTNU, Trondheim, Norway

**Correction: Behavior Research Methods (2024) 56:3330-3345**

10.3758/s13428-024-02408-1

The original online version of this article was revised. The Funding Source was updated to: The research leading to these results has received funding from the Norwegian Financial Mechanism 2014-2021, no. 2019/34/H/HS6/00677.

