# Supplementary material for: Development and validation of the Emotional Climate Change Stories (ECCS) stimuli set
Source: Behav Res Methods. 2024 Apr 18;56(4):3330–45. doi: 10.3758/s13428-024-02408-1 (PMC11133034; doi:10.3758/s13428-024-02408-1)
Supplement: Supplementary file 1 — Supplementary file1 (DOCX 658 KB) [file 13428_2024_2408_MOESM1_ESM.docx]

**Table S1.** *Story length (number of characters) for each language version of ECCS*

| ECCS version | M | SD | Range |
| --- | --- | --- | --- |
|  |  |  |  |
| ECCS - Polish | 307.2 | 44.49 | 192-408 |
| ECCS - Norwegian | 295.2 | 49.35 | 169-421 |
| ECCS - English | 304.0 | 51.65 | 176-424 |

**Table S2.** *Demographic profile of the study participants.*

|  | Study 1 | Study 2 | Study 3 |
| --- | --- | --- | --- |
| N total | 601 | 307 | 346 |
| Gender | 504 F, 92 M, 5 other | 164 F, 142 M, 1 other | 181 F, 165 M |
| Age | M = 26.26 (range 18-62) | M = 39.09 (range 18-73) | M = 45.57 (range 18-85) |
| Place of residence | | | |
| Rural | 11% | 25% | 5% |
| Urban under 50 000 | 13% | 25% | 45% |
| Urban over 50 000 - 100 000 | 9% | 14% | 24% |
| Urban over 100 000 | 66% | 36% | 25% |
| Education | | | |
| Primary | 0.5% | 11% | 8% |
| Secondary | 59% | 62% | 45% |
| Higher | 41% | 26% | 48% |
| Belief in climate change: Climate change… | | | |
| … is definitely happening | 83% | 59% | 53% |
| … is likely happening | 14% | 36% | 41% |
| … is likely not happening | 1% | 4% | 6% |
| … is definitely not happening | 2% | <1% | <1% |
| Concern about climate change | | | |
| High | 25% | 32% | 28% |
| Medium | 44% | 35% | 36% |
| Low | 29% | 32% | 36% |
| Other factors influencing climate change attitudes | | | |
| Parents | 16% | 56% | 62% |
| Employment related to climate change * | 7% | 10% | 27% |
| Climate activists | 6% | 11% | 35% |

*) e.g. researcher, NGO, public services, or private sector related to climate change or environmental protection

**Table S3**. *General information about the collected ratings*

|  | Study 1 | Study 2 | Study 3 |
| --- | --- | --- | --- |
| Total number of ratings | 86499 | 42833 | 56700 |
| Number of stories rated per participant | M = 20.56, SD = 15.65, range 10-179 | M = 19.93, SD = 16.91, range 10-130 | M = 23.41, SD = 24.48, range 10-180 |

**Table S4**. *Differences between ratings of stories of each type on their respective scales between Study 1 and Study 2. In each case, comparisons for the scales that were relevant for a given story type are underscored.*

| ANOVA posthoc: Ratings of ANG stories (Study 1 vs Study 2) | | | | | | | |
| --- | --- | --- | --- | --- | --- | --- | --- |
| **scale** | **group1** | **group2** | **estimate** | **conf.low** | **conf.high** | **p.adj** | **p.adj.signif** |
| anger | Study 1 | Study 2 | -3.59 | -9.26 | 2.07 | .642 | ns |
| anxiety | Study 1 | Study 2 | 9.87 | 4.1 | 15.63 | <.0001 | **** |
| compassion | Study 1 | Study 2 | 14.31 | 8.78 | 19.84 | <.0001 | **** |
| guilt | Study 1 | Study 2 | 16.58 | 11.03 | 22.14 | <.0001 | **** |
| hope | Study 1 | Study 2 | 20.9 | 15.82 | 25.99 | <.0001 | **** |

| ANOVA posthoc: Ratings of ANX stories (Study 1 vs Study 2) | | | | | | | |
| --- | --- | --- | --- | --- | --- | --- | --- |
| **scale** | **group1** | **group2** | **estimate** | **conf.low** | **conf.high** | **p.adj** | **p.adj.signif** |
| anger | Study 1 | Study 2 | 5.5 | -0.27 | 11.26 | .0785 | ns |
| anxiety | Study 1 | Study 2 | -2.77 | -8.64 | 3.1 | .929 | ns |
| compassion | Study 1 | Study 2 | 0.75 | -4.88 | 6.37 | 1 | ns |
| guilt | Study 1 | Study 2 | 10.11 | 4.46 | 15.77 | <.0001 | **** |
| hope | Study 1 | Study 2 | 21.87 | 16.7 | 27.05 | <.0001 | **** |

| ANOVA posthoc: Ratings of COM stories (Study 1 vs Study 2) | | | | | | | |
| --- | --- | --- | --- | --- | --- | --- | --- |
| **scale** | **group1** | **group2** | **estimate** | **conf.low** | **conf.high** | **p.adj** | **p.adj.signif** |
| anger | Study 1 | Study 2 | 2.96 | -2.79 | 8.71 | .876 | ns |
| anxiety | Study 1 | Study 2 | 3.13 | -2.73 | 8.98 | .846 | ns |
| compassion | Study 1 | Study 2 | -4.9 | -10.51 | 0.71 | .157 | ns |
| guilt | Study 1 | Study 2 | 8.62 | 2.97 | 14.26 | <.0001 | **** |
| hope | Study 1 | Study 2 | 22.7 | 17.53 | 27.86 | <.0001 | **** |

| ANOVA posthoc: Ratings of GUI stories (Study 1 vs Study 2) | | | | | | | |
| --- | --- | --- | --- | --- | --- | --- | --- |
| **scale** | **group1** | **group2** | **estimate** | **conf.low** | **conf.high** | **p.adj** | **p.adj.signif** |
| anger | Study 1 | Study 2 | 8.05 | 2.33 | 13.77 | .00027 | *** |
| anxiety | Study 1 | Study 2 | 8.22 | 2.4 | 14.04 | .00025 | *** |
| compassion | Study 1 | Study 2 | 10.9 | 5.32 | 16.48 | <.0001 | **** |
| guilt | Study 1 | Study 2 | 5.42 | -0.19 | 11.03 | .0697 | ns |
| hope | Study 1 | Study 2 | 18.78 | 13.65 | 23.92 | <.0001 | **** |

| ANOVA posthoc: Ratings of HOP stories (Study 1 vs Study 2) | | | | | | | |
| --- | --- | --- | --- | --- | --- | --- | --- |
| **scale** | **group1** | **group2** | **estimate** | **conf.low** | **conf.high** | **p.adj** | **p.adj.signif** |
| anger | Study 1 | Study 2 | 15.38 | 9.72 | 21.04 | <.0001 | **** |
| anxiety | Study 1 | Study 2 | 16.27 | 10.51 | 22.03 | <.0001 | **** |
| compassion | Study 1 | Study 2 | 16.72 | 11.19 | 22.24 | <.0001 | **** |
| guilt | Study 1 | Study 2 | 15.51 | 9.95 | 21.06 | <.0001 | **** |
| hope | Study 1 | Study 2 | -1.65 | -6.73 | 3.44 | .996 | ns |

**Table S5**. *Differences between ratings of stories of each type on their respective scales between Study 2 and Study 3. In each case, comparisons for the scales that were relevant for a given story type are underscored.*

| ANOVA posthoc: Ratings of ANG stories (Study 2 vs Study 3) | | | | | | | |
| --- | --- | --- | --- | --- | --- | --- | --- |
| **part** | **group1** | **group2** | **estimate** | **conf.low** | **conf.high** | **p.adj** | **p.adj.signif** |
| anger | Study 2 | Study 3 | -1.49 | -8.12 | 5.14 | 1 | ns |
| anxiety | Study 2 | Study 3 | 6.94 | 0.24 | 13.64 | .0347 | * |
| compassion | Study 2 | Study 3 | 4.2 | -2.21 | 10.6 | .592 | ns |
| guilt | Study 2 | Study 3 | 6.81 | -0.1 | 13.72 | .0579 | ns |
| hope | Study 2 | Study 3 | 6.32 | -0.53 | 13.18 | .104 | ns |

| ANOVA posthoc: Ratings of ANX stories (Study 2 vs Study 3) | | | | | | | |
| --- | --- | --- | --- | --- | --- | --- | --- |
| **part** | **group1** | **group2** | **estimate** | **conf.low** | **conf.high** | **p.adj** | **p.adj.signif** |
| anger | Study 2 | Study 3 | 0.87 | -5.85 | 7.59 | 1 | ns |
| anxiety | Study 2 | Study 3 | 0.84 | -5.96 | 7.63 | 1 | ns |
| compassion | Study 2 | Study 3 | 1.45 | -5.05 | 7.94 | 1 | ns |
| guilt | Study 2 | Study 3 | 3.73 | -3.28 | 10.73 | .85 | ns |
| hope | Study 2 | Study 3 | 6.14 | -0.81 | 13.09 | .145 | ns |

| ANOVA posthoc: Ratings of COM stories (Study 2 vs Study 3) | | | | | | | |
| --- | --- | --- | --- | --- | --- | --- | --- |
| **part** | **group1** | **group2** | **estimate** | **conf.low** | **conf.high** | **p.adj** | **p.adj.signif** |
| anger | Study 2 | Study 3 | -0.27 | -6.97 | 6.43 | 1 | ns |
| anxiety | Study 2 | Study 3 | 1.24 | -5.53 | 8.02 | 1 | ns |
| compassion | Study 2 | Study 3 | -3.41 | -9.88 | 3.07 | .859 | ns |
| guilt | Study 2 | Study 3 | 4.36 | -2.62 | 11.35 | .664 | ns |
| hope | Study 2 | Study 3 | 6.62 | -0.31 | 13.55 | .0773 | ns |

| ANOVA posthoc: Ratings of GUI stories (Study 2 vs Study 3) | | | | | | | |
| --- | --- | --- | --- | --- | --- | --- | --- |
| **part** | **group1** | **group2** | **estimate** | **conf.low** | **conf.high** | **p.adj** | **p.adj.signif** |
| anger | Study 2 | Study 3 | 2.07 | -4.6 | 8.74 | .997 | ns |
| anxiety | Study 2 | Study 3 | 5.34 | -1.4 | 12.08 | .286 | ns |
| compassion | Study 2 | Study 3 | 7.17 | 0.73 | 13.61 | .0146 | * |
| guilt | Study 2 | Study 3 | 5.36 | -1.59 | 12.32 | .325 | ns |
| hope | Study 2 | Study 3 | 6.24 | -0.65 | 13.14 | .121 | ns |

| ANOVA posthoc: Ratings of HOP stories (Study 2 vs Study 3) | | | | | | | |
| --- | --- | --- | --- | --- | --- | --- | --- |
| **part** | **group1** | **group2** | **estimate** | **conf.low** | **conf.high** | **p.adj** | **p.adj.signif** |
| anger | Study 2 | Study 3 | 12.39 | 5.78 | 19 | <.0001 | **** |
| anxiety | Study 2 | Study 3 | 9.94 | 3.25 | 16.63 | <.0001 | **** |
| compassion | Study 2 | Study 3 | 17.44 | 11.05 | 23.83 | <.0001 | **** |
| guilt | Study 2 | Study 3 | 9.41 | 2.52 | 16.31 | .00051 | *** |
| hope | Study 2 | Study 3 | -5.79 | -12.63 | 1.05 | .194 | ns |

**Figure S1**. *Comparisons of ratings of each story between Study 1 (opportunity sample) and Study 2 (purposive sample).*


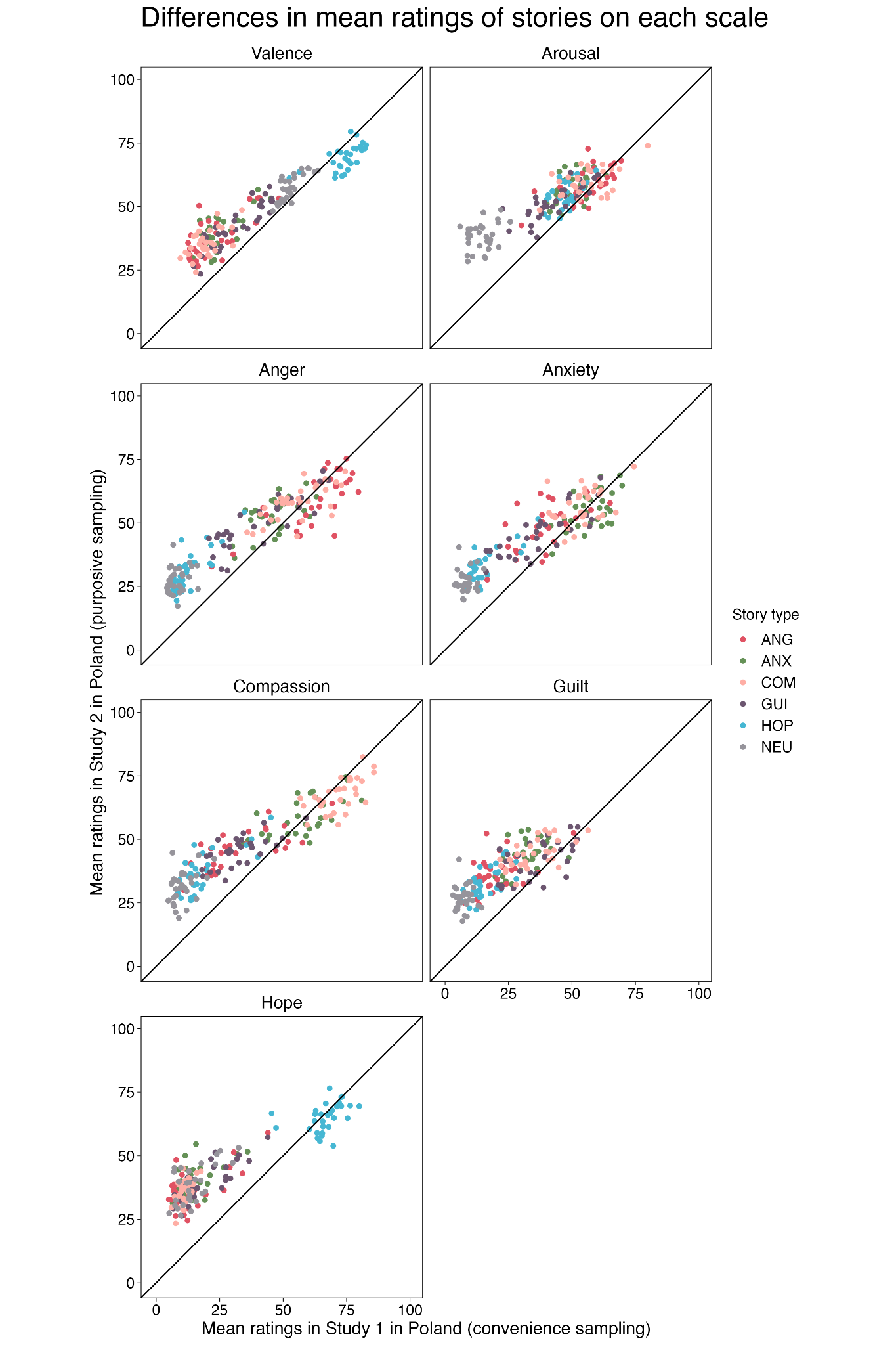


Note: Individual stories are represented by dots. Colours denote different story types: ANG - anger, ANX - anxiety, COM - compassion, GUI - guilt, HOP - hope, NEU - neutral.

**Figure S2**. *Comparisons of ratings of each story between Study 2 (Polish sample) and Study 3 (Norwegian sample).*


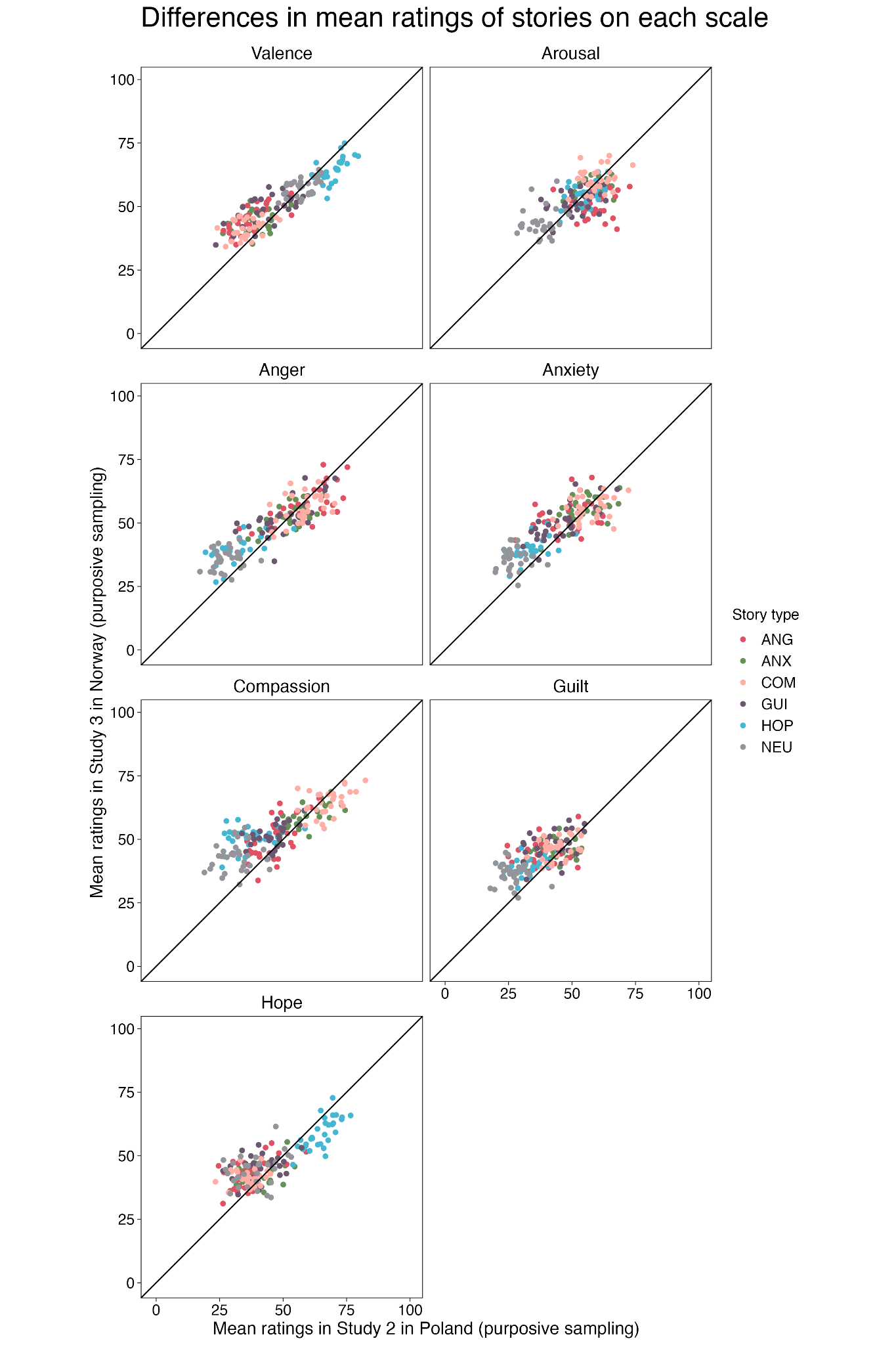


Note: Individual stories are represented by dots. Colours denote different story types: ANG - anger, ANX - anxiety, COM - compassion, GUI - guilt, HOP - hope, NEU - neutral.

**Figure S3**. *Results of the classification analysis for different threshold values. The figure illustrates how class sizes change as we modify the criteria for a story to be assigned to one of the classes (from the most liberal: 0, to the most conservative: 140).*


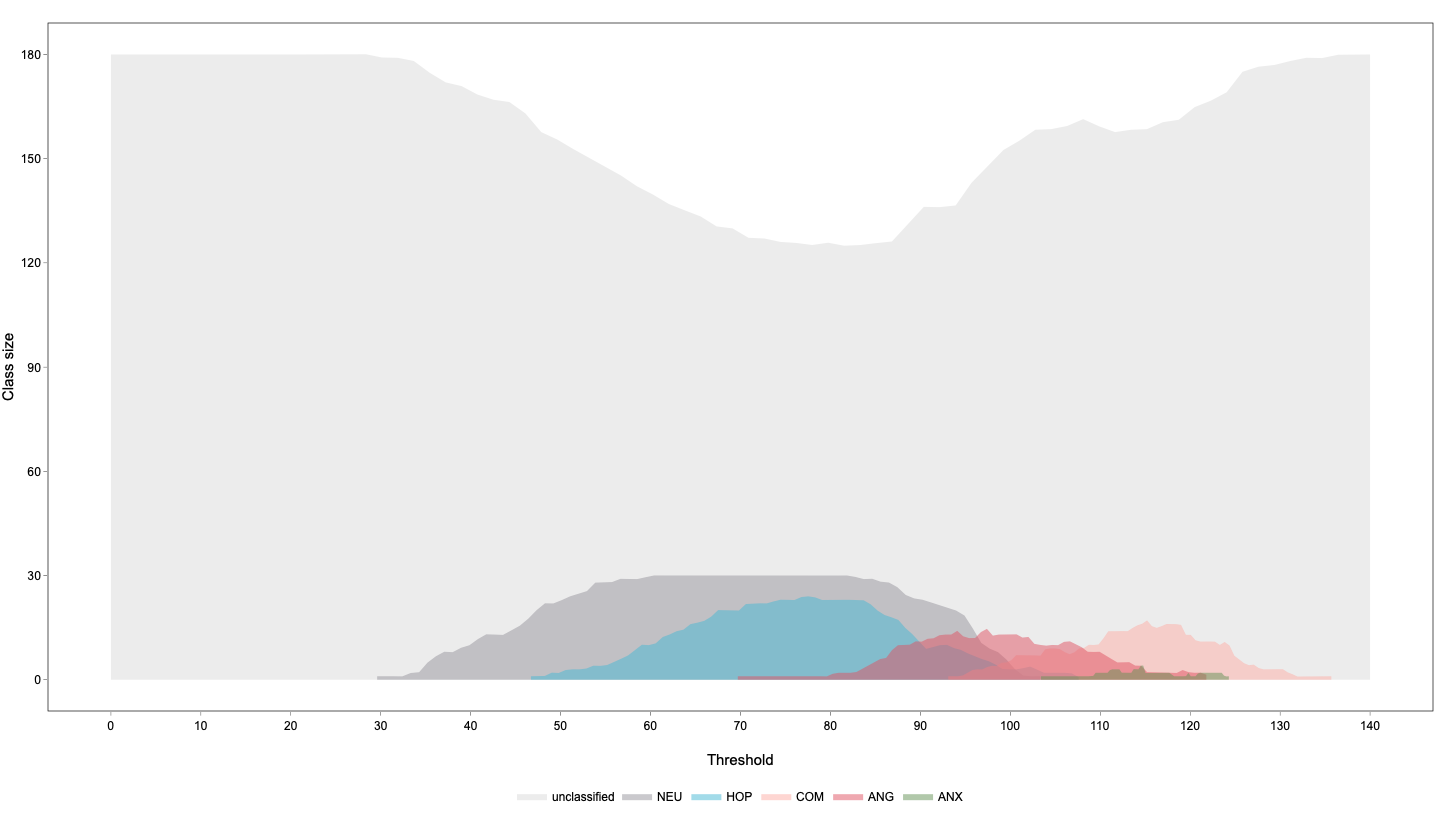


Note: Colours denote different classes: NEU - neutral, HOP - hope, COM - compassion, ANG - anger, ANX - anxiety. Unclassified stories are marked with light gray for visualization purposes.
